# Make Vitamin D While the Sun Shines, Take Supplements When It Doesn′t: A Longitudinal, Observational Study of Older Adults in Tasmania, Australia

**DOI:** 10.1371/journal.pone.0059063

**Published:** 2013-03-18

**Authors:** Jane K. Pittaway, Kiran D. K. Ahuja, Jeffrey M. Beckett, Marie-Louise Bird, Iain K. Robertson, Madeleine J. Ball

**Affiliations:** School of Human Life Sciences, University of Tasmania, Launceston, Tasmania, Australia; Oklahoma State University, United States of America

## Abstract

Low vitamin D status has been associated with a number of chronic conditions, particularly in older adults. The aim of this study was to identify how best to maintain optimum vitamin D status throughout the year in this high-risk population. The main objectives of the study were to assess seasonal vitamin D status; identify the main determinants of vitamin D status; determine if taking part in the study led to alterations in participant behaviour and vitamin D status. A longitudinal design across four consecutive seasons observed ninety-one 60–85 year old community-dwelling adults in Tasmania (41π S) over 13 consecutive months, with a follow-up assessment at next winter's end. Associations between solar UVB exposure, sun protection behaviours, dietary and supplemental vitamin D with serum 25(OH)D concentrations were assessed. Variation in serum 25(OH)D demonstrated an identical pattern to solar UVB, lagging 8–10 weeks. Serum 25(OH)D was positively associated with summer UVB (mean 15.9 nmol/L; 95%CI 11.8–19.9 nmol/L, p<0.001) and vitamin D supplementation (100–600 IU/day: 95%CI 10.2 nmol/L; 0.8–19.6 nmol/L; p = 0.03; 800 IU/day: 21.0 nmol/L; 95%CI 8.1–34.0 nmol/L; p = 0.001). Seasonal variation in serum 25(OH)D was greatly diminished in supplement users. The most common alteration in participant behaviour after the study was ingesting vitamin D supplements. Post-study vitamin D supplementation ℘800 IU/day was seven times more likely than during the study resulting in mean difference in serum 25(OH)D between supplement and non-supplement users of 30.1 nmol/L (95%CI 19.4–40.8 nmol/L; p<0.001). The main limitation was homogeneity of participant ethnicity. Solar exposure in summer and ingestion of vitamin D supplements in other seasons are the most effective ways of achieving and maintaining year-round vitamin D sufficiency in older adults in the Southern hemisphere. Vitamin D supplementation has greatest effect on vitamin D status if ingested during and after winter, i.e. between the autumn and spring equinoxes.

## Introduction

In Australia, as in many countries with ageing populations, research into preventative health in older adults has become a priority because institutionalisation of the elderly is a major cost burden. [1] The relationship between vitamin D and a number of medical conditions has been well researched during the past decade, [2], [3], [4], [5] suggesting associations of varying significance between vitamin D status and: bone mineral density, osteoporosis, bone fractures, lower limb strength, balance and falls, multiple sclerosis, Type 1 diabetes, other autoimmune diseases, common cancers, cardiovascular disease, kidney disease, chronic and acute infections and mental illness.

The physiological indicator of vitamin D status is serum 25(OH)D, a product of normal vitamin D synthesis in the body. A recent consensus of expert opinions [2], [4], [6], [7] suggested the concentration of serum 25(OH)D deemed sufficient for normal, healthy function is at least 50 nmol/L, with concentrations below 25 nmol/L considered deficient. There is some debate however about what constitutes optimal vitamin D status. Some authors consider 75 nmol/L reflects optimal status, [4], [6], [8] whereas others do not consider there is consistently increased benefit at this higher concentration. [2], [7]

Cutaneous exposure to UVB via sunlight is the greatest contributor to vitamin D status. [4], [9], [10], [11] Availability of sunlight varies with the seasons, more so at higher latitudes compared to at the equator, because of both the total amount of UVB available and the angle at which solar radiation hits the earth's surface. Thus, serum 25(OH)D concentration may also show seasonal variation, especially at higher latitudes.

Determining a ‘safe’ amount of sun exposure for optimum cutaneous vitamin D synthesis without increasing skin cancer risk is not straightforward [12], [13] and depends on a number of factors such as time of year, latitude and skin pigmentation.[4], [11] Furthermore, cutaneous synthesis of vitamin D diminishes with age, [9], [14], [15] yet natural dietary sources of vitamin D are few [4], [5], [16] and their contribution to vitamin D status is minimal. [17], [18], [19], [20] Nevertheless, recent research [15], [21], [22], [23], [24], [25] suggests ageing populations, especially those with limited sunlight exposure, have a greater dependency on dietary sources to maintain optimal vitamin D status, be it through increased consumption of vitamin D-rich foods, food fortification or vitamin D supplementation.

Some researchers believe there is a world-wide ‘epidemic’ of vitamin D deficiency, [6], [26] particularly in older age groups. A UK study (57π N) of postmenopausal women [17] found that in winter, 50–75% of the Western European group were vitamin D insufficient (25(OH)D<40 nmol/L), with 10–40% vitamin D deficient (25(OH)D<25 nmol/L). In summer, 9% were still vitamin D deficient. A Dutch study (52π N) of older men and women, [27] reported that 51% of their participants had winter serum 25(OH)D concentrations less than 50 nmol/L and in a Canadian study (45π N), [24] 5.7% of men and 1.9% of women were deemed vitamin D deficient after summer.

This study extends research into determinants of vitamin D status in community-dwelling older women and men, by employing a longitudinal, observational design across four consecutive seasons in a Southern Hemisphere location (41π S). Additionally, participants were re-assessed at the end of the following winter, nine months after the study had finished and study restrictions were lifted. Post study follow-up to ascertain the effect of study participation is not a common component of research designs.

Previous research was either cross-sectional in design, [15], [21], [22], [23], [24] or did not assess participants over consecutive seasons. [25] The strength of the longitudinal design of the current study is that it is less sensitive to variation within the study population (and thus within a wider population) because it focuses on changes within each study participant. [28] Much of the previous research has been undertaken in the Northern hemisphere at higher latitudes (>45π N), [21], [22], [23], [24], [25] whereas in the Southern Hemisphere, the majority of the population live at latitudes between 45π south and the equator. The participants of the current study lived in and around Launceston, Tasmania (41π S). The current study invited older men and women to participate, whereas three of the five previous studies focused on a single gender. [15], [22], [23]

The aim of this study was to identify how best to maintain participants' optimum vitamin D status throughout the year. The specific objectives were: to assess the seasonal vitamin D status; to identify the main determinants of vitamin D status, and finally to determine if taking part in the study led to alterations in participant behaviour and vitamin D status.

## Materials and Methods

### Ethics statement

This study was conducted according to the guidelines laid down in the Declaration of Helsinki and was approved by the Human Research Ethics Committee (Tasmania, Australia) Network (HREC), reference number H0010561. Written, informed consent was obtained from all participants and they were free to leave the study at any time.

### Study design setting and participants

This longitudinal, observational study was part of a larger study investigating the likely determinants of seasonal variation in balance in older, community-dwelling Tasmanian adults. [29] The study was undertaken during a 15 month period, from the end of winter (September) 2009 to the end of spring (December) 2010, in and around Launceston, Tasmania, Australia, latitude 41π south. The balance measure chosen was medio-lateral sway range, which is the total distance in a sideways direction that the centre of mass moves in set time frame (30 seconds) as measured with a force platform in a standing position. *A-priori* sample size calculation for the larger study based on medio-lateral sway range suggested a minimum requirement of 81 completed participants (minimum effect size 2.5 mm sway; SD 8 mm; power 0.8, alpha 0.05): 109 were enrolled with the anticipation of a 15% drop-out rate. *Post hoc* analysis suggested that this number might allow the detection of a minimum change in serum 25(OH)D of 8.0 nmol/L between subjects ingesting vitamin D supplements compared to those not taking supplements, assuming a standard deviation of change in serum 25(OH)D of 11.7 nmol/L and 25% of the subjects taking vitamin D supplements with a power of 0.8.

Adults aged between 60 and 85 years were invited to participate via local media (print and radio) and local community meetings. Recruitment took place over an 11-week period, between the end of August and the beginning of November 2009. Participants were self-selected, independent living, community-dwelling Tasmanians who were able to ambulate independently inside, without the use of walking aids. Exclusion criteria comprised recent or current acute medical conditions, or an uncontrolled chronic condition, clinically diagnosed kidney or liver disease, taking prescription medication that interfered with vitamin D activity and taking oral vitamin D supplements greater than 800 IU/day. To test the logistics and recruitment techniques of the study, a small group of participants commenced at the end of winter (September) 2009, these participants completed the initial study at the end of Winter 2010. The majority commenced at the end of Spring (December) 2009 and completed at the end of Spring 2010. Data was collected for each participant over a consecutive 13-month period. Participants were assessed on five occasions timed to coincide with expected seasonal fluctuations in vitamin D status based on daily solar exposure. Participants were also invited to attend a follow-up visit nine months after the final study time point.

At each study appointment, participants had measurements to calculate body mass index (BMI) and body fat mass and a small sample of venous blood drawn, which was centrifuged and the serum separated and frozen for subsequent serum 25(OH)D analysis. They were surveyed on habitual dietary intake, outdoor activity and sun avoidance behaviour during the previous three months using standard questionnaires. Subsequent appointments were booked, three months in advance, to coincide with the end of the next season. Participants received their serum 25(OH)D results after their final study appointment. At the follow-up appointment, participants were also asked about any changes in relevant behaviours that resulted as a consequence of them participating in the study. They received their follow-up serum 25(OH)D result soon afterwards.

### Variables measured

Concentration of serum 25(OH)D was measured by a nationally accredited diagnostic pathology laboratory, using the DiaSorin LIAISON method (DiaSorin Inc, Stillwater, MN, USA), which employs competitive chemiluminescent immunoassay (CLIA) technology. The laboratory participates in the Royal College of Pathology Australia external Quality Assurance program (RCPA-QAP) for serum 25(OH)D analysis. All samples for each participant were analysed in the same batch to reduce inter assay variation. The intra assay coefficient of variation was 8% at a level of 38 nmol/L and 6% at a level of 132 nmol/L. The sensitivity of the method, as stated by the manufacturers, was 10 nmol/L. The clinical thresholds for serum 25(OH)D concentration in Table 1 were chosen to encompass the recommendations in the current literature. [2], [4], [6], [7], [30]

**Table 1:** Relative proportions of participants with serum 25(OH)D concentrations (nmol/L) below different clinical thresholds.^1^

Vitamin D (nmol/L)			<25 nmol/L			<50 nmol/L			<75 nmol/L		
Time period^2^	n^3^	Mean^3^±SD	n (%)	IRR^4^ (95% CI)	p	n (%)	IRR (95% CI)	p	n (%)	IRR (95% CI)	p
Winter 2009	18	49.0±17.0	2 (11)	1.77 (0.20 to 15.40)	0.61	10 (56)	0.95 (0.61 to 1.47)	0.81	16 (89)	0.97 (0.84 to 1.13)	>0.90
Spring 2009	90	60.5±18.4	1 (1)	0.12 (0.02 to 0.98)	0.19	29 (32)	0.66 (0.53 to 0.82)	<0.001	73 (81)	0.95 (0.87 to 1.01)	>0.90
Summer 2010	91	68.7±21.1	2 (2)	0.24 (0.06 to 0.96)	0.26	17 (19)	0.38 (0.25 to 0.59)	<0.001	63 (69)	0.82 (0.72 to 0.92)	0.01
Autumn 2010	88	58.8±21.5	2 (2)	0.25 (0.06 to 0.99)	0.15	35 (40)	0.81 (0.65 to 1.01)	0.13	70 (80)	0.94 (0.87 to 1.01)	0.31
Winter 2010	86	52.2±21.0	8 (9)	1.00		42 (49)	1.00		73 (85)	1.00	
Spring 2010	72	58.9±20.0	3 (4)	0.42 (0.13 to 1.33)	0.28	26 (36)	0.77 (0.63 to 0.95)	0.04	60 (83)	1.00 (0.93 to 1.08)	>0.90
Winter 2011	72	68.8±18.7	1 (1)	0.15 (0.02 to 0.98)	0.24	10 (14)	0.28 (0.15 to 0.51)	<0.001	46 (64)	0.75 (0.62 to 0.90)	0.01

1 Clinical thresholds were chosen to encompass the recommendations in the current literature [2] [4] [7] [30] [6]. <25 nmol/L = deficient, 25–50 nmol/L = insufficient, 50–75 nmol/L = sufficient, >75 nmol/L = optimal.

2 Measurements for each time period were made over three weeks at the end of each season. “Winter 2011” represented the follow-up period, nine months after completion of the primary study, and after the participants were released from restricting vitamin D supplementation (study exclusion criterion>800 IU/day).

3 Number of subjects assessed at each time period are shown, and mean serum 25(OH)D concentration (±standard deviation) were estimated by mixed methods linear regression, adjusted for participant age and time from beginning of study. Large variation in participant numbers (Winter 2009, Spring 2010) is because a small group of participants commenced the study at the end of Winter (September) 2009 and completed at the end of Winter 2010; the majority commenced at the end of Spring (December) 2009 and completed at the end of Spring 2010.

4 The relative proportion of participants having serum 25(OH)D concentration below different clinical thresholds shown was compared to the relative proportion at the end of Winter (September) 2010 (chosen as the references because the end of Winter 2009 represented the pilot group only), estimated using repeated-measures negative binomial regression and expressed as an incidence rate ratio (IRR; 95% confidence intervals; p-values).

Bodyweight and body fat mass were assessed using bio-impedance scales (Tania, BF-522W, Tokyo, Japan). Dual-energy x-ray absorptiometry total body scans were not employed as the study researchers were travelling between testing sites and needed portable equipment. Dietary vitamin D intake during the previous three months was assessed using a 113-item semi-quantitative food frequency questionnaire (FFQ) that contained 33 specific food items known to be good sources of vitamin D, for example, Atlantic salmon, canned sardines and other oily fish, eggs, cream and other dairy foods. Although FFQs have their limitations, the same FFQ was used throughout the study primarily to observe any changes in individual dietary intake. Dietary data was analysed using Foodworks 2009 dietary analysis software (version 6, Xyris, Brisbane, Australia), incorporating the AusNut and Nuttab95 databases. Ingestion of supplements containing vitamin D was recorded separately to dietary intake.

Cutaneous sun exposure was assessed using a recall questionnaire developed by the study researchers, based on the format of the Community Healthy Activities Model Program for Seniors (CHAMPS) questionnaire. [31] Participants recalled time spent outside in a typical week during the previous four weeks and sun avoidance behaviours were recorded for the same time period. Sun avoidance behaviours included frequency (always, sometimes, never) of sunscreen use, wearing a hat, wearing protective clothing and staying out of the sun between 10.00 am and 3.00 pm. Information about daily solar exposure (representing solar UVB exposure) for Launceston for the whole of the study period was obtained from the Australian Bureau of Meteorology (http://www.bom.gov.au/tas/observations/index.shtml).

### Statistical analysis

Serum 25(OH)D results were first grouped by five individual collection time points to identify any variation that may have occurred during the study and any relationship with solar UVB exposure. Data was then organised into four seasonal groupings, to enable investigation of the determinants of vitamin D status based on season rather than of specific time-points. This meant that the seasonal groupings for winter and spring included data from two time points (i.e. September 2009 and September 2010 for winter and December 2009 and December 2010 for spring). The associations between serum 25(OH)D concentrations at end of winter compared to other seasons, taking vitamin D supplements, wearing protective clothing, and percentage body fat mass were estimated using repeated-measures mixed methods linear regression analysis adjusted for age and time of subject visit from beginning of study on 1^st^ September 2009. Variables for inclusion in this model were selected using stepwise regression from: age, gender, sun exposure, sun avoidance, use of sunscreen, wearing hat, wearing protective clothing, weight, vitamin D supplements, dietary vitamin D, dietary fat as percentage of energy, saturated fat as percentage of total fat.

The relative proportions of participants with sub-threshold serum vitamin D concentrations was estimated using repeated-measures negative binomial regression and expressed as an incidence rate ratio (IRR; 95% confidence intervals (CI); p-values). Serum vitamin D concentration data was fitted to a sine-wave model using repeated-measures seemingly unrelated non-linear regression, adjusted for age, and mean concentration, the amplitude and timing of the annual cycle of change estimated and compared between participants not taking supplements and those taking supplements. P-values were corrected where appropriate for multiple comparisons using the Holm method. All statistical analyses were performed using STATA SE12.1, (Statacorp, College Station TX, USA). PRISM 5.03 (GraphPad Software Inc., La Jolla CA, USA) was used for graphic presentation of variation in serum 25(OH)D concentration (median, 25%, 75% ranges; mean, 95%CI).

Trial registration: Australia and New Zealand Clinical Trials Registry (ANZCTR): ACTRN12612000159853.

## Results

### Participants

One hundred and nine participants were initially enrolled in the study. Five participants chose not to follow through after enrolment, eight could not attend appointments at the scheduled times, one withdrew before their first appointment due to illness, two withdrew after their first appointment due to time commitments and two withdrew after their second appointment due to health issues. Data from 91 participants, who attended three or more appointments, were included in the study analysis: 65 females (71%) and 26 males (29%). Mean (±SD) age was 69.4±6.5 years, BMI 27.5±4.0 kg/m^2^ and body fat mass 34.0±10.2%. Of these 91 participants, one participant passed away between the end of the study period and follow-up, one participant withdrew and nine did not reply to correspondence (two were overseas at the time of correspondence). Thus 81 participants supplied data at follow-up, 58 females (72%) and 23 males (28%). Nine participants could not attend follow-up appointments for blood collection but completed questionnaires, thus 72 blood samples were included in the follow-up serum 25(OH)D analysis: 50 females (69%) and 22 males (31%).

### Seasonal vitamin D status

**Figure 1:**
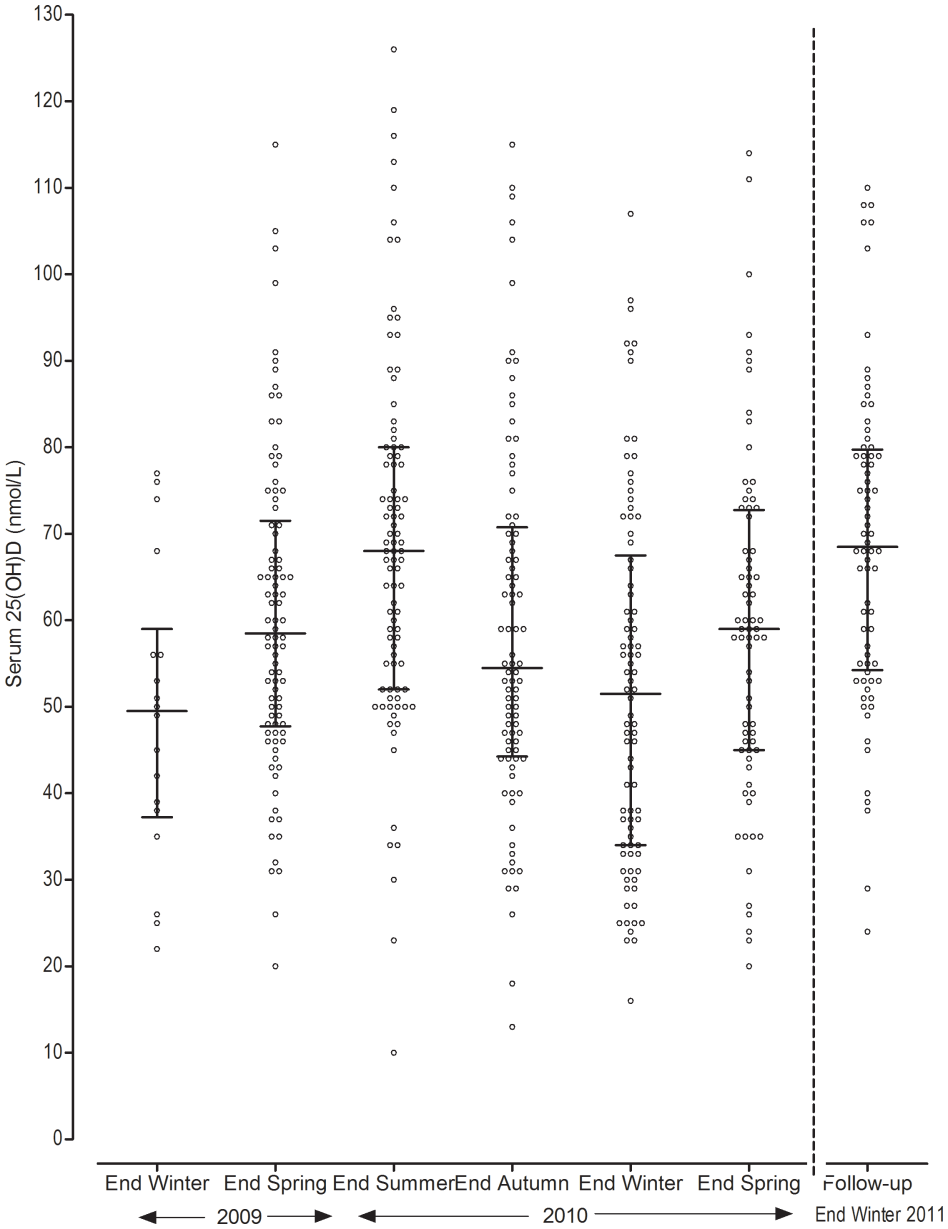
Participant serum 25(OH)D concentrations at each time-point during the study and at follow-up. ^1^Circles represent individual participant serum 25(OH)D concentrations (nmol/L). ^2^Lines and cross-bars represent the median value at each time point with 25% and 75% interquartile ranges.

**Figure 2:**
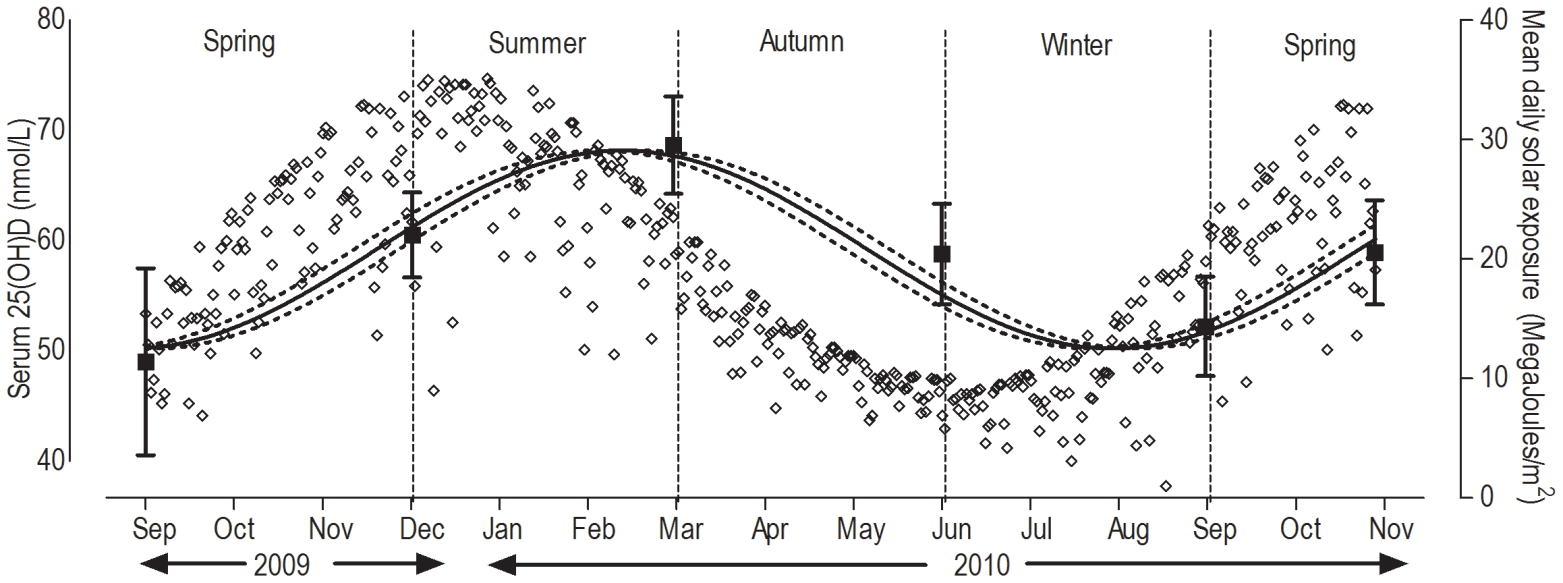
Relationship between mean daily solar exposure and serum 25(OH)D concentrations during the study. ^1^Diamonds represent mean daily solar exposure during the study measured as Mega joules/m^2^. Data obtained from the Australian Bureau of Meteorology (http://www.bom.gov.au/tas/observations/index.shtml). ^2^Square points and y-axis error bars represent mean and 95% confidence intervals of serum 25(OH)D concentrations (nmol/L) measured at each study time point estimated by repeated-measures mixed methods linear regression; and sign-wave lines were calculated by mathematical modelling using coefficients for sine-wave analysis estimated using repeated-measures nonlinear regression.

Variation in serum 25(OH)D concentration (median, 25%, 75% ranges) followed a seasonal cycle (Figure 1). The lowest median serum 25(OH)D concentrations (49.5 nmol/L; 51.5 nmol/L) occurred at the end of the winter collection periods (2009 and 2010, respectively) and the highest, 68.0 nmol/L, at the end of summer. The median serum 25(OH)D concentration for the follow-up, timed for the end of winter 2011, was 68.5 nmol/L. Graphical representation of mean daily solar exposure (representing solar UVB exposure) during the study (Figure 2) displayed a seasonal, sigmoidal pattern. Mathematical modelling of mean serum 25(OH)D concentration, applying a sine wave model, demonstrated an identical pattern to mean daily solar exposure but with an 8–10 week time lag, i.e. mean daily solar exposure peak at ∼36 Mega joules/m^2^ in summer (January) followed by 25(OH)D peak at the end of summer (March; mean 68.7 nmol/L; 95%CI 64.3 to 74.1 nmol/L) and mean daily solar exposure trough in winter (June, ∼8 Mega joules/m^2^) followed by 25(OH)D trough at the end of winter (August; mean 52.2 nmol/L; 95%CI 47.7 to 56.7 nmol/L).

Relative proportions (Table 1) of the participants with serum 25(OH)D concentrations below the clinically significant threshold value of 50 nmol/L (sufficiency), [2], [4], [6], [7], [30] were significantly lower (p<0.001) at the end of spring 2009 and 2010 (32% & 36%), compared to the end of winter 2010 (49%). Moreover, the relative proportion of participants with serum 25(OH)D concentrations below 50 nmol/L was even fewer at the end of summer 2010 (19%). This relative proportion was also significantly lower (p<0.001) compared to the end of winter 2010 (49%). At the follow-up period, only 14% of participants had serum 25(OH)D concentration less than 50 nmol/L, again a significantly lower relative proportion of participants compared to the end of winter 2010 (49%)(p<0.001). At the serum 25(OH)D concentration threshold of 75 nmol/L (optimal), [4], [6], [30] there was a significant difference in relative proportions of participants less than this threshold only between the end of summer compared to the end of winter 2010 (69% vs. 85%; p = 0.01). At the follow-up appointments, 64% of participants had a serum 25(OH)D concentration less than 75 nmol/L, again significantly lower than the relative proportion of participants under this threshold at end of winter 2010 (85%)(p = 0.01).

### Determinants of vitamin D status in the initial study

Selection of measured variables associated with vitamin D status by stepwise regression (Table 2) showed significant positive association of serum 25(OH)D concentration with vitamin D supplementation (100–600 IU/day p = 0.03; 800 IU/day p = 0.001) and seasonal solar UVB exposure (during spring, autumn and summer; all p<0.001). Body fat mass (p = 0.02) and the use of protective clothing (p = 0.007) showed significant negative association with serum 25(OH)D concentration. Determinants not selected into the final stepwise regression model were vitamin D contribution from diet (p = 0.71), time spent outside (p = 0.49) and the other sun avoidance behaviours measured: wearing a hat (p = 0.21), using sunscreen (p = 0.57) and avoiding exposure between 10.00 am and 3.00 pm (p = 0.77).

**Table 2:** The most significant determinants of vitamin D status (serum 25(OH)D concentration, nmol/L).

Determinants	Mean^1^(nmol/L)	95% CI (nmol/L)	
Seasonal solar exposure: Winter^2^	50.9	(41.8 to 60.0)	
Seasonal solar exposure:	Δ from mean^1^	95% CI	p-value
Spring^2^	5.4	(2.9 to 7.9)	<0.001
Summer^2^	15.9	(11.8 to 19.9)	<0.001
Autumn^2^	5.8	(3.0 to 8.6)	<0.001
Vitamin D supplements:			
(100–600 IU/day)	10.2	(0.8 to 19.6)	0.03
(800 IU/day)	21.0	(8.1 to 34.0)	0.001
Wearing protective clothing^3^	−5.4	(−10.3 to −0.5)	0.03
Body fat mass (%)^3^	−4.2	(−8.8 to −0.8)	0.02

1 Mean serum 25(OH)D concentration (nmol/L) at end of Winter, the mean difference (95% confidence intervals; p-values) at the end of other seasons, and the effect of taking Vitamin D supplements, wearing protective clothing, and percentage body fat mass, were estimated using repeated-measures mixed methods linear regression analysis adjusted for age and time of subject visit. Variables for inclusion in this model were selected using stepwise regression from: Age, gender, sun exposure, sun avoidance, use of sunscreen, wearing hat, wearing protective clothing, weight, vitamin D supplements, dietary vitamin D, dietary fat as percentage of energy, saturated fat as percentage of total fat.

2 Data was organised into four seasonal groupings, to enable investigation of the determinants of vitamin D status based on season rather than of specific time-points.

3 The effect of wearing protective clothing and percentage body fat mass (as standardised normal transformations) on serum 25(OH)D concentration was expressed as the slope of the association: one standard deviation rise in each measure was associated with change shown in the table (95% confidence intervals of the slope; p-values).

Vitamin D contribution from diet did not vary significantly during the study. The greatest intake occurred during winter, with a mean (±SD) of 4.52±2.12 µg/day and the least during autumn (4.41±1.70 µg/day), a difference of 0.23 µg/day (95%CI−0.51 to 0.04 µg/day, p = 1.00). Dairy foods contributed most dietary vitamin D (2.11 µg/day) with non-dairy spreads (0.87 µg/day), fresh seafood (0.73 µg/day) and canned fish (0.29 µg/day) being the other main contributors.

Twenty five per cent of the study participants used vitamin D supplements (<800 IU/day) during the study period (excluding follow-up). There was no significant change in the proportion of people or the amount of supplement intake between seasons (Table 3). Participants who ingested vitamin D supplements had significantly higher serum 25(OH)D concentrations than those who did not for all seasons of the study except at the end of summer (Table 4). The greatest mean difference (22.8 nmol/L; 95%CI: 8.7 to 23.0 nmol/L; p<0.001) in serum 25(OH)D concentration was at the end of winter between participants taking 800 IU/day vitamin D and those who were not taking any supplements. At the end of summer, the mean difference between those ingesting 800 IU/day and non-supplemented participants was 4.8 nmol/L (p>0.90). The mean amplitude of seasonal variation in serum 25(OH)D concentration (Table 5) for non-supplemented participants throughout the study was 10.6±9.1 nmol/L whereas for participants ingesting 800 IU/day it was 0.2±1.9 nmol/L, a mean difference of 10.5 nmol/L (95%CI: 5.6 to 15.4 nmol/L; p<0.001).

**Table 3:** Relative proportions of participants ingesting vitamin D supplements during the study and at follow-up.

	n^1^	Relative proportion (%)	IRR^2^ (95% CI)	p-value
Any supplements				
Winter	26 of 104	(25.0%)	1.00	
Spring	40 of 162	(24.7%)	0.95 (0.80 to 1.10)	0.36
Summer	22 of 91	(24.2%)	0.94 (0.90 to 1.00)	0.24
Autumn	22 of 88	(25.0%)	0.93 (0.80 to 1.10)	0.27
Follow-up^3^	53 of 81	(65.4%)	2.48 (1.80 to 3.50)	<0.001
800 IU/day				
Winter	7 of 104	(6.7%)	1.00	
Spring	15 of 162	(9.3%)	1.22 (0.80 to 1.80)	0.33
Summer	7 of 91	(7.7%)	1.06 (0.70 to 1.50)	0.77
Autumn	6 of 88	(6.8%)	0.93 (0.70 to 1.20)	0.62
Follow-up^3^	43 of 81	(53.1%)	7.43 (3.6 to 15.5)	<0.001

1 Seasonal groupings for winter and spring included data from two time points (i.e. September 2009 and September 2010 for winter and December 2009 and December 2010 for spring).

2 The relative proportion of participants taking Vitamin D supplements of different doses at the end of different seasons was compared to the relative proportion at the end of Winter (September), estimated using repeated-measures negative binomial regression and expressed as an incidence rate ratio (IRR; 95% confidence intervals; P-values).

3 Follow-up: at the end of Winter (September) 2011, nine months after the final study time point and after participant were released from restricting vitamin D supplementation (study exclusion criterion was>800 IU/day).

**Table 4:** Effect of vitamin D supplementation on serum 25(OH)D concentrations (nmol/L) during the study and at follow-up.

	No supplements		Vitamin D supplements		Comparison^1^		
	n^2^	Mean^1^±SD (nmol/L)	n	Mean^1^±SD (nmol/L)	Difference	95%CI	p-value
No supplements compared to Vitamin D supplements (100–600 IU/day)							
Winter	78	46.5±19.0	19	62.4±18.6	15.9	(8.7 to 23.0)	<0.001
Spring	122	55.6±18.7	25	64.0±18.8	8.4	(2.1 to 14.8)	0.07
Summer	69	66.9±22.3	15	68.8±18.0	1.8	(−5.4 to 9.0)	>0.90
Autumn	66	54.3±20.6	16	66.8±19.8	12.6	(5.4 to 19.7)	0.01
Follow-up	24	51.3±16.1	10	60.0±12.0	8.7	(−1.7 to 19.0)	0.18
No supplements compared to Vitamin D supplements (≥800 IU/day)^3^							
Winter	78	46.5±19.0	7	69.3±19.0	22.8	(13.0 to 32.5)	<0.001
Spring	122	55.6±18.7	15	71.6±14.9	16.0	(6.9 to 25.1)	0.002
Summer	69	66.9±22.3	7	71.8±19.6	4.8	(−5.8 to 15.4)	>0.90
Autumn	66	54.3±20.6	6	71.5±21.9	17.2	(6.4 to 28.1)	0.01
Follow-up	24	51.3±16.1	38	81.4±17.5	30.1	(19.4 to 40.8)	<0.001

1 Mean (±standard deviation) and comparison of serum 25(OH)D concentration (nmol/L) in patients with and without (low- and high-dose) supplements at the end of different seasons, estimated by repeated-measures mixed methods linear regression (mean difference; 95% confidence intervals; P-values corrected for multiple comparisons by the Holm method), adjusted for age and time of subject visit.

2 Large variation in participant numbers (Winter 2009, Spring 2010) is because a small group of participants commenced the study at the end of Winter (September) 2009 and completed at the end of Winter 2010; the majority commenced at the end of Spring (December) 2009 and completed at the end of Spring 2010.

3 Maximum allowable dose of vitamin D supplements increased between the end of the study and the follow-up appointment.

**Table 5:** Amplitude of annual variation in serum 25(OH)D concentrations (nmol/L) in different supplementation groups.

	n	Mean^1^±SD (nmol/L)	Mean amplitude^2^±SD	Δ of mean amplitudes^3^	95% CI	p-value
No supplement	68	56.8±17.0	10.6±9.1	–		
100–600 IU/day	17	65.6±14.9	5.3±7.4	5.3	(0.5 to 10.0)	0.03
800 IU/day	6	71.5±17.2	0.2±1.9	10.5	(5.6 to 15.4)	<0.001

1 Mean amplitude of annual variation, estimated by repeated measures non-linear regression using a sine wave model, adjusted for age and gender.

2 Mean difference of amplitude between 100–600 IU/day and 800 IU/day: 5.2 (95%CI-1.6 to 12.0; p = 0.13).

3 Comparison of the mean amplitude between non-supplement and supplement groups.

### Changes in behaviour and vitamin D status at follow-up

Participants did not report significant changes in diet or sun avoidance/exposure behaviours. In contrast, participants were more than twice as likely to be ingesting a vitamin D supplement (Table 3) at follow-up (i.e. when study restrictions of supplementation≤800 IU/day were removed), than during the study (IRR 2.48; 95%CI 1.80 to 3.50; p<0.001) and over seven times more likely to be taking≥800 IU/day (IRR 7.43; 95%CI 3.60 to 15.50; p<0.001). The mean difference in serum 25(OH)D concentration for participants taking≥800 IU/day at follow up (end winter 2011) compared to those not taking supplements (Table 4) was 30.1 nmol/L (95%CI: 19.4 to 40.8 nmol/L; p<0.001).

## Discussion

Variation in mean serum 25(OH)D concentrations (representing vitamin D status) for the whole group followed a cyclic, seasonal pattern across the study time-points, lagging 8–10 weeks behind the peaks and troughs of seasonal solar exposure (representing solar UVB exposure). Even so, the seasonal pattern of serum 25(OH)D concentrations was attenuated in participants who ingested vitamin D supplements. For participants taking supplements of 800 IU/day, the amplitude in seasonal variation was almost completely diminished, with mean winter serum 25(OH)D concentrations very close to summer levels. Nine months after the study, at the end of winter follow-up, ingesting a vitamin D supplement was the most significant change noted in participant behaviour. Participants were more than twice as likely to be taking any vitamin D supplement and more than seven times more likely to be taking supplements≥800 IU/day. Participants ingesting the higher dosage revealed mean serum 25(OH)D concentrations 59% greater than non-supplemented participants at follow-up and 13% greater than the highest summer study levels.

In the initial study, 9% of the study participants at the end of winter were deemed vitamin D deficient (serum 25(OH)D <25 nmol/L), 49% were vitamin D insufficient (serum 25(OH)D<50 nmol/L) and 85% had serum 25(OH)D concentrations less than 75 nmol/L (sufficient but not optimal). The variety of clinical cut-offs used in the literature made direct comparison of vitamin D status difficult. Even so, studies on similar populations using similar clinical cut-offs have reported similar results. [17], [24], [27] In contrast, a previous Australian study (38–39π S) [32] and a New Zealand study (37π S), [15] reported up to double the incidence of vitamin D deficiency (<25 nmol/L) in their participants during winter (17.6% and 6–16%, respectively compared to 9% in the current study) and up to 25% greater incidence of insufficiency (<50 nmol/L), than the current study (60.3% and 56–74% compared to 49%). The previous Australian study population differed from the current study in that it comprised women presenting at hospital after having had a fall, compared to the ambulatory men and women of the current study. While the New Zealand study specified vitamin D supplementation of ℘1000 IU/day (or ℘ 50,000 IU/month) in their exclusion criteria, the authors did not report the proportion of participants taking supplements and whether their vitamin D status was different from those not taking supplements. In the current study, approximately 25% of participants consumed vitamin D supplements and up to 9% were taking 800 IU/day, which may have contributed to their vitamin D status.

The time lag between seasonal solar UVB exposure and serum 25(OH)D concentrations observed in the current study was found to be eight to ten weeks i.e. about two weeks longer than that observed by previous studies.[15], [21], [32], [33] This difference may be due to a number of potential mitigating factors including: variation in sun avoidance behaviours, the amount of solar UVB available for vitamin D synthesis (dependant on local weather, pollution, ozone concentration,[33] latitude, and season), and the age range of study populations.

Summer solar UVB exposure and vitamin D supplementation were positively associated with vitamin D status while body fat mass and use of protective clothing were negatively associated with vitamin D status. These results were generally consistent with those identified in previous studies. [15], [17], [27], [34], [35] In contrast, Hill *et.al*. [36] found no association between sun avoidance and serum 25(OH)D concentration. This difference in results may be due to the timing of when information on behaviour i.e. sun avoidance was collected. While in the current study participants were asked to recall previous four weeks of outdoor activity prior to each study appointment, Hill and colleagues collected this information at the next visit, which was six months later.

An association between dietary vitamin D and serum 25(OH)D concentration, not observed in the current study, has been reported in previous studies. [19], [22], [23], [37] Participants in these studies were from countries such as Sweden, where eating oily fish and food fortification is much more prevalent than in Australia. [35], [38] Although dietary vitamin D intake in the current study (4.41±1.70 to 4.52±2.12 µg/day) was more than twice that of a cohort in a previous Australian study [33] it was well below the Australian and New Zealand nutrient reference value (15 ug/day) for the age range of the study population. [38] In Australia, margarine is the only food mandated to be fortified with vitamin D, although some other foodstuffs such as skim milk, yoghurt and cheese are voluntarily fortified [38] as are some breakfast cereals. Dairy products contributed most to the dietary vitamin D intake of the current study; non-dairy spreads (margarine) were the next best contributing food group.

A previous Australian multi-centre, cross-sectional study published in 2007, [33] found no association between vitamin D supplementation and serum 25(OH)D concentration in their Tasmanian cohort. Eight per cent of their participants recorded supplement usage, compared to 25% in the current study. The 423 female and 299 male participants were all less than 60 years old. The higher proportion of supplement users observed in the current study may be due to the increased public awareness of vitamin D research in Australia during the past few years. Additionally, older individuals may be more likely to be heeding health advice and taking vitamin D supplements.

The current study confirms observations of two previous studies, [21], [24] regarding continuance of summer vitamin D sufficiency into winter, in participants taking 800 IU of vitamin D per day. Even so, both of these studies were cross-sectional, assessing different individuals at different time points. Results from the follow-up appointment, at the end of the winter after the current study, adds further evidence regarding the efficacy of vitamin D supplementation to diminish seasonal variation and maintain optimal vitamin D status year round. This effect of supplement on serum 25(OH)D concentrations, suggests supplementation during summer may be less necessary than during the other seasons except in individuals who received very little solar exposure due to cultural practices [39] or adherence to ‘sun safe’ messages. [12], [40] These findings are however in contrast to a previous longitudinal study in Irish post-menopausal women [25] which reported a significant difference in serum 25(OH)D concentrations between ‘supplement users’ and ‘non-users’ at all three measured time-points-February/March (end of winter), August/September (end of summer) and February/March the following year.

We observed a 25% variation in vitamin D status between seasons of low and high UVB exposure. This seasonal influence should be considered in the clinical setting when interpreting serum 25(OH)D as a sufficient result at the end of winter may be a better indicator of adequacy throughout the year. However, sufficient but sub-optimal status during/at the end of summer would potentially be of concern due to the significant reductions in serum vitamin D from summer to winter, as observed in the current study. When planning vitamin D supplementation for maximum effect, the time lag between peak solar exposure and maximum vitamin D status should be considered. For example, in Tasmania the best time for supplementation appears to be from the end of summer to the end of winter.

The strengths of this study were the longitudinal design, inclusion of a follow-up appointment and the *ad libitum* observational approach, allowing ‘real world’ assessment. Data collection from the same group of participants during consecutive seasons accounted for inter-personal variation and the inclusion of a follow-up appointment, allowed the study researchers to ascertain the effect of changes in participant behaviour, because of taking part in the study and receiving their study vitamin D result, on subsequent vitamin D status. Homogeneity of participant ethnicity was the main limitation of the study; all were white, of western European descent. Thus, no inferences can be made about populations of differing ethnicity or skin type. Further research is needed to determine optimal vitamin D status to help prevent chronic diseases and quantify the vitamin D supplementation required to achieve this status.

## Conclusions

Solar exposure in summer and ingestion of vitamin D supplements in other seasons are the most effective ways of achieving and maintaining year-round vitamin D sufficiency in older adults in the Southern hemisphere. Vitamin D supplementation has greatest effect on vitamin D status if ingested during and after winter, i.e. between the autumn and spring equinoxes.
